# Contribution of KCTD12 to esophageal squamous cell carcinoma

**DOI:** 10.1186/s12885-018-4765-z

**Published:** 2018-08-29

**Authors:** Mohammad Reza Abbaszadegan, Negin Taghehchian, Liping Li, Azadeh Aarabi, Meysam Moghbeli

**Affiliations:** 1 Immunology Research Center, Mashhad University of Medical Sciences, Mashhad, Iran; 2 Medical Genetics Research Center, Faculty of Medical Sciences, Mashhad University of Medical Sciences, Mashhad, Iran; 3 Department of Clinical Laboratory, The Third Affiliated Hospital of Nanchang University, Jiangxi, Nanchang 330008 People’s Republic of China; 4 Department of Modern Sciences and Technologies, Faculty of Medicine, Mashhad University of Medical Sciences, Mashhad, Iran

**Keywords:** Self-renewal, Chromatin remodeling, Esophageal cancer, NOTCH, WNT, Stem cell

## Abstract

**Background:**

It has been shown that the expression of potassium channel tetramerization domain containing 12 (KCTD12) as a regulator of GABAB receptor signaling is reversely associated with gastrointestinal stromal tumors. In present study we examined the probable role of KCTD12 in regulation of several signaling pathways and chromatin remodelers in esophageal squamous cell carcinoma (ESCC).

**Methods:**

KCTD12 ectopic expression was done in KYSE30 cell line. Comparative quantitative real time PCR was used to assess the expression of stem cell factors and several factors belonging to the WNT/NOTCH and chromatin remodeling in transfected cells in comparison with non-transfected cells.

**Results:**

We observed that the KCTD12 significantly down regulated expression of NANOG, SOX2, SALL4, KLF4, MAML1, PYGO2, BMI1, BRG1, MSI1, MEIS1, EGFR, DIDO1, ABCC4, ABCG2, and CRIPTO1 in transfected cells in comparison with non-transfected cells. Migration assay showed a significant decrease in cell movement in ectopic expressed cells in comparison with non-transfected cells (*p* = 0.02). Moreover, KCTD12 significantly decreased the 5FU resistance in transfected cells (*p* = 0.01).

**Conclusions:**

KCTD12 may exert its inhibitory role in ESCC through the suppression of WNT /NOTCH, stem cell factors, and chromatin remodelers and can be introduced as an efficient therapeutic marker.

## Background

Esophageal cancer is the sixth leading cause of cancer related deaths in the world [1]. Squamous cell carcinoma (ESCC) and adenocarcinoma are the main subtypes of esophageal cancer which are common in developing and developed countries, respectively. ESCC involves more than 95% of esophageal cancers in Asia [2]. ESCC has a hot spot in Asian Esophageal Cancer Belt spreading from the China to Caspian Sea [3]. Despite the novel chemoradiotherapeutic modalities, ESCC has still a five-year survival below 20% because of the late diagnosis in advanced stages of tumor [4, 5]. It has been shown that deregulation of cellular signaling pathways such as WNT, NOTCH, SHH, and BMP is extensively involved in ESCC progression and drug resistance [6–10]. Therefore, targeting such pathways can be efficient in paving the way of targeted therapy in such patients. There is not any reported of a single marker to cover and regulate all of the mentioned pathways in esophageal cancer. Potassium channels regulate a wide spectrum of cellular processes through potassium flow across cell membranes. Cancer constitutes a category of channelopathies disorder highlighting the probable role of potassium channels in cell proliferation. KCTD12 (Potassium Channel Tetramerization Domain Containing 12) is auxiliary subunit of GABA-B receptors which alter the G-protein signaling of the receptors. Its expression is observed in different fetal organs such as cochlea and brain, however, it has low levels of expression in adult tissues [11]. It is involved in stabilizing and up regulation of GABAB receptors [12]. Moreover, KCTD12 can be a prognostic factor of gastrointestinal stromal tumors (GISTs) [13]. KCTD12 facilitates M phase entrance and promote cancer cell proliferation which is done by CDK1 dephosphorylation by KCTD12. Therefore, KCTD12, CDK1, and CDC25B complex play an important role in tumor cell cycle regulation [14]. KCTD12 regulates self-renewal and drug resistance, through the ERK signaling pathway [15]. Colorectal cancer stem cells have also shown a down regulation of KCTD12 which is a differentiation factor in relation with ERK pathway [15]. There is a controversy in KCTD12 function in which, KCTD12 plays as an oncogene in gastrointestinal stromal tumors; [16] and as a tumor suppressor in colon cancer [15]. KCTD12 is also involved in cell cycle regulation through its interaction with CDK1 and CDC25B [14]. In addition, KCTD 21, 11, and 6, have been reported to regulate the proliferation of medulloblastoma stem cells via the HDAC1 and sonic hedgehog signaling pathway [17, 18]. Epigenetic abnormalities such as changes in signaling pathways and chromatin remodeling have been shown as common characteristics for specific cancers. Notch signaling pathway has been assessed during embryonic development and self-renewal of adult organs. It functions through cell-to-cell contact in the regulation of tissue homeostasis and stem cell maintenance [19, 20]. Deregulation of Notch pathway has been reported in a variety of malignancies [21–23]. Regarding the expression patterns, it can function either oncogenic or tumor suppressive through regulation of cell proliferation, arrest, and differentiation [24]. WNT signaling pathway is also another important regulatory pathway in embryonic development, cell cycle regulation, and cancer [9]. It has been shown that tumor progression is related to the epigenetic and genomic changes [25]. The vital processes such as DNA synthesis, repair, and transcription are regulated by dynamic changes in nucleosome structure which is significantly involved in DNA-binding proteins access to DNA [26]. Therefore, it is inevitable that aberrations in chromatin remodelers are correlated with tumor progression [27, 28]. Homeoproteins are also key components of regulatory pathways which are involved in both organogenesis and oncogenesis. They function as transcription factors in normal tissues through activation or inhibition of their target genes. Therefore aberrant expression of HOX family members can be critical for tumorigenesis, indicating the role of such components in tissue homeostasis [6, 10]. In present study we assessed for the first time a probable correlation between KCTD12 as a K^+^ ion channel component and other epigenetic processes such as NOTCH/WNT pathways, chromatin remodelers, and HOX genes which are the main oncogenic factors in esophageal cancer. This study was performed to introduce the KCTD12 as a master regulator of chromatin remodeling and signaling pathways during ESCC progression.

## Methods

### Cell culture and transfection

**Fig. 1:**
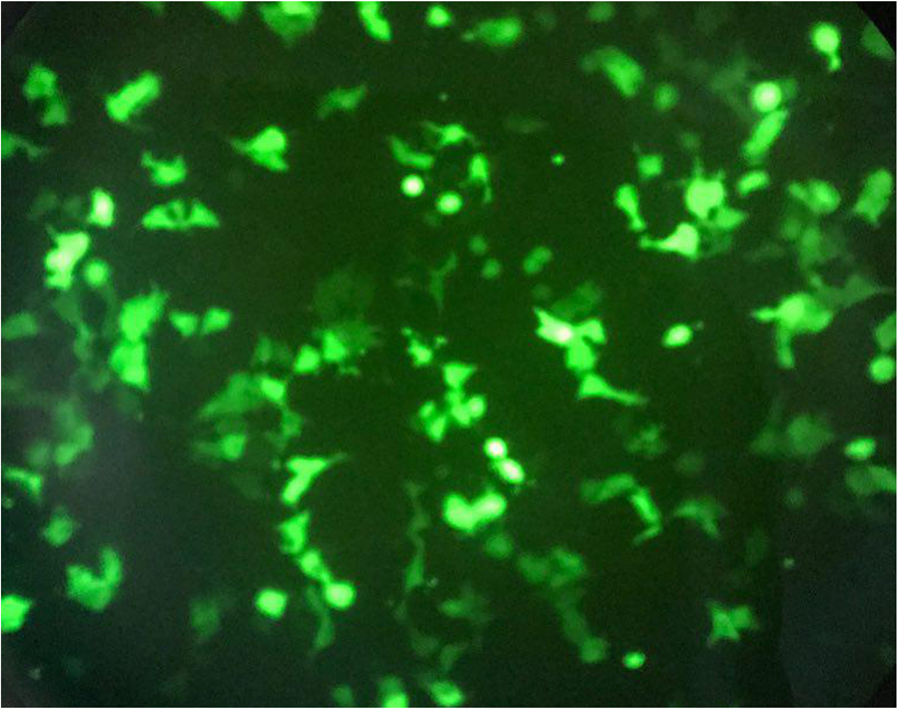
KCTD12 ectopic expression and GFP expression using X-treme GENE HP DNA Transfection Reagent

We used KCTD12- pbabe for ectopic expression in KYSE30 cell line [15]. KYSE30 cell lines (Pasteur Institute, Iran) were cultured in a serum free RPMI1640 medium (5% CO2, 37 °C). Transfection was performed using the X-treme GENE HP DNA (Roche. Germany). All the of the transfections were performed in 6 wells plates 24 h. following the cell culture (4 × 10^5^ seeded cells per well) according to manufactures protocol. pB-GFP vector was used as control of transfection (Fig. 1).

### Migration assay and drug resistance

ESCC cells were seeded in 6-well plate, cultured until 90–100% confluent for 24 h, and transfected with KCTD12-pbabe in a serum free media as described above. Monolayers were scratched using a 200 μl pipette tip, and washed with PBS to eliminate the detached cells. Wound closure was checked and images were captured at 0, 24 and 48 h (Optica, Italy). Percentage of wound closure was analyzed using Image J software (1.42 version, national institute of health, USA). All the migration assays were repeated three times. MTT assay was done in tetra-plicate reactions for the 5-Fluorouracil (5-FU) resistance, in which 3 × 10^4^ cells were seeded per well and cultured overnight. The cells were treated with 12.5 μg/ml of 5FU for 48 h. The plates were quantified at an absorbance of 570 nm.

### cDNA synthesis and quantitative RT-PCR

RNA extraction and cDNA synthesis from transfected KYSE30 cell line were performed using the RNeasy Mini kit (Qiagen, Hilden, Germany) and first-strand synthesis kit (Fermentas, Lithuania), respectively. Subsequently, quantitative SYBR green (BIORON, Germany) RT-PCR was done in duplicate reactions (LightCycler, Roche, Germany). Glyceraldehyd 3-phosphat dehydrogenase (GAPDH) was used as a normalizer [29]. All the primer sequences and thermal profiles are mentioned in Table 1. Gene expression was analyzed using 2- ΔΔCT algorithm. Relative expression levels and fold changes were log2 transformed for data-analysis. More than one-fold of fluorescence intensity in transfected cells in comparison with non-transfected cells was considered as over expression. Less than − 1 fold indicated under expression.

**Table 1:** Primer sequences and thermal profiles

Genes	Sequence (5’to 3’)	Amplification Size(bp)
GAPDH	F: GGAAGGTGAAGGTCGGAGTCA	101 bp
	R:GTCATTGATGGCAACAATATCCACT	
TWIST1	F: GGAGTCCGCAGTCTTACGAG	201 bp
	R:TCTGGAGGACCTGGTAGAGG	
BMI1	F: CGTGTATTGTTCGTTACCTGGAGAC	204 bp
	R:CATTGGCAGCATCAGCAGAAGG	
Cripto	F: GGGATACAGCACAGTAAGGAG	295 bp
	R:ACGGTGGTAGTTGTCGAGTC	
KLF4	F:TCTTCTCTTCGTTGACTTTG	210 bp
	R:GCCAGCGGTTATTCGG	
Nanog	F: GGCAATGGTGTGACGCAGAAGGC	137 bp
	R:GCTCCAGGTTGAATTGTTCCAGGTC	
MSI1	F: TGAGCAGTTTGGGAAGGTG	117 bp
	R: TCACACACTTTCTCCACGATG	
MEIS1	F: ATGACACGGCATCTACTCGTTC	105 bp
	R: TGTCCAAGCCATCACCTTGCT	
SOX2	F: AACAGCCCGGACCGCGTCAA	189 bp
	R: TCGCAGCCGCTTAGCCTCGT	
MAML1	F: TCTCGCGGAACAGGAGA	123 bp
	R: GCAGCAGAGGACCCTGTG	
PYGO2	F: GTCCCCCACTCCATGGCCGCCTCG	147 bp
	R: TCGCTTCTTTTCTGGACTCTTC	
EGFR	F: ACCGGCAGGATGTGGAGATC	186 bp
	R: GGCCGACAGCTATGAGATGGA	
ABCG2	F: TGAGGGTTTGGAACTGTGG	155 bp
	R: GATTCTGACGCACACCTGG	
ABCC4	F: GAAATTGGACTTCACGATTTAAGG	125 bp
	R: TTCCACAGTTCCTCATCCGT	
DIDO1	F: TTTGTTGGTCCAGTTTCGCCTTC	220 bp
	R: ACGACAAGCAAGAGACTGTTTCAC	
SMARCA4	F: TGTGAGGAGGAGGAGGAGAA	164 bp
	R: CGCTTCCGTGATGATTTCTT	

## Results

### Stem cell factors

**Fig. 2:**
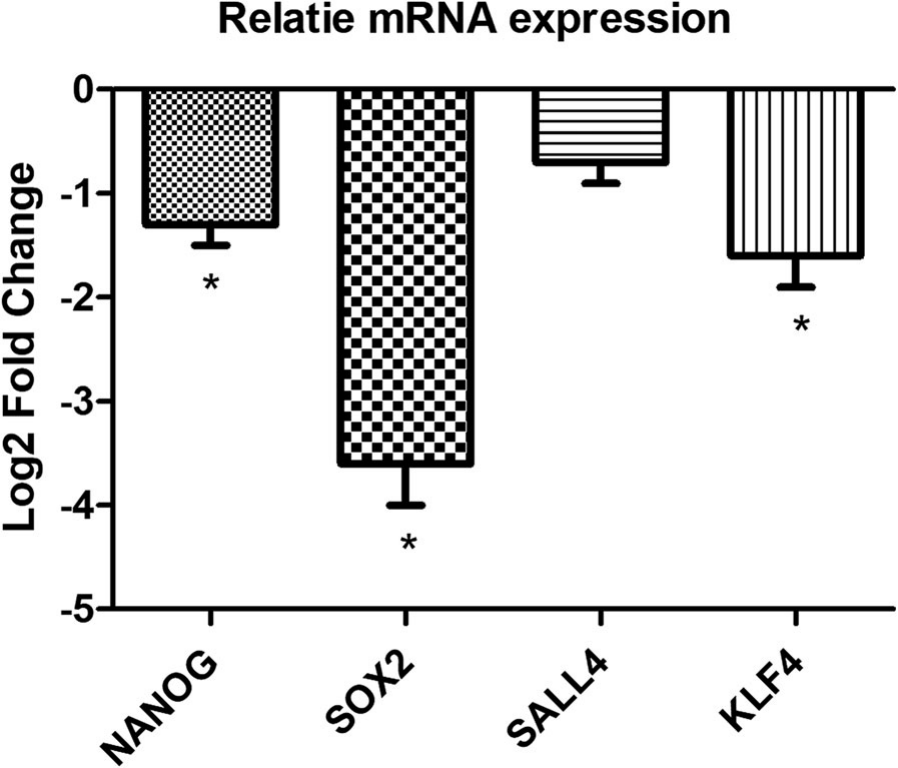
Relative mRNA expression of stem cell factors in KCTD12 transfected in comparison with non-transfected cells

Regarding the role of self-renewal factors such as SOX2, NANOG, KLF4, and SALL4 in biology of tumor cells we assessed the probable role of KCTD12 in regulation of such factors in the levels of mRNA expression. It was shown that the KCTD12 significantly down-regulated the expression of SOX2, NANOG, and KLF4 with − 3.6, − 1.3, and − 1.6 fold changes respectively in transfected cells in comparison with non-transfected cells (Fig. 1). Whereas, there wasn’t any significant down regulation in the case of SALL4 (− 0.7 fold changes) (Fig. 2).

### NOTCH and WNT signaling factors

**Fig. 3:**
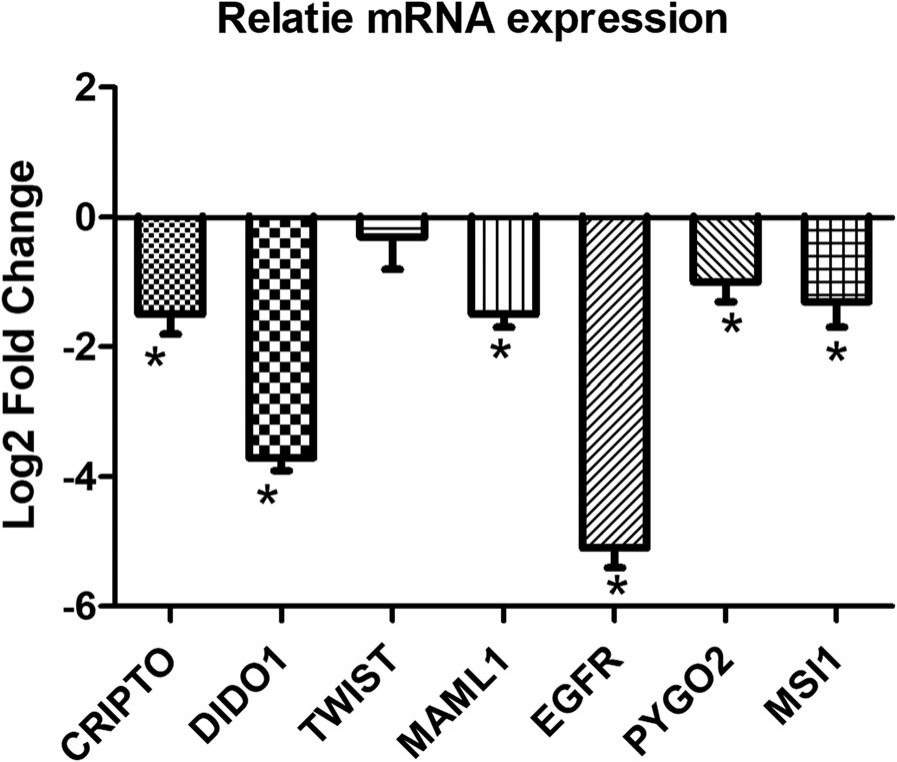
Relative mRNA expression of WNT and NOTCH signaling factors in KCTD12 transfected in comparison with non-transfected cells

Beside the stem cell factors, we assessed the probable correlation between KCTD12 expression and NOTCH pathway through the expressional analysis for several components of this pathway. MAML1 as the main components of NOTCH transcription machinery was assessed in the levels of mRNA expression in KCTD transfected cells in comparison with the non-transfected cells. The results showed that there was a significant correlation between KCTD12 and MAML1 in which the levels of MAML1 in transfected cells were lower than that in non-transfected cells (− 1.5 fold changes). We also analyzed the expression of several factors such as HES1 and HEY1 as the main target genes in NOTCH pathway and interestingly there wasn’t any significant correlation between KCTD12 and these two markers (0.48 and − 0.01 fold changes, respectively). In the case of WNT pathway we have also assessed the levels of PYGO2 as the main member of WNT transcription machinery and it was shown that there was a significant under expression in KCTD12 transfected cells in comparison with the non-transfected cells (− 1.00 fold changes). MSI1 and CRIPTO1 as the main positive regulators of WNT and NOTCH pathways were also evaluated in transfected cells. We observed a significant under expression of MSI1 and CRIPTO1 in KCTD12 transfected cells in comparison with non-transfected cells (− 1.3 and − 1.5 fold changes, respectively). EGFR and DIDO1 also as WNT target genes had significant under expression (− 5.1 and − 3.7 fold changes, respectively) (Fig. 3).

### Chromatin remodeling and HOX factors

**Fig. 4:**
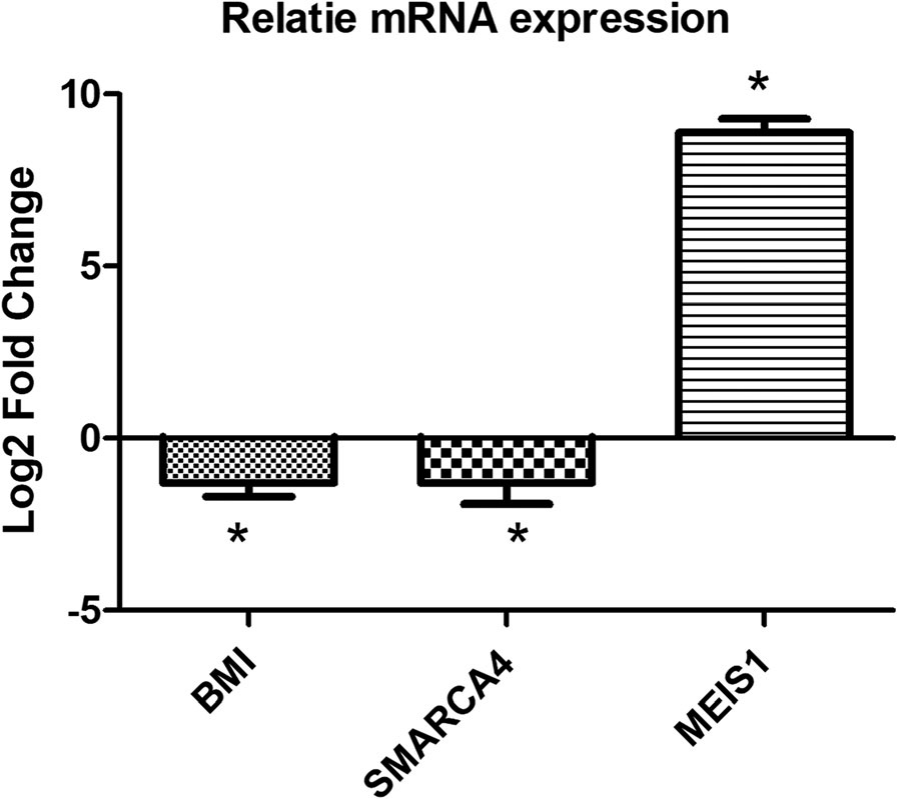
Relative mRNA expression of chromatin remodeling factors in KCTD12 transfected in comparison with non-transfected cells

Indeed, access to DNA structure is an essential step in epigenetic regulation. Therefore, we assessed the expression of SMARCA4 and BMI1 as the hall marks of chromatin remodeling regulation. Interestingly, we observed a significant negative correlation between KCTD12, SMARCA4, and BMI1 in which the KCTD12 down regulated the SMARCA4 and BMI1 in transfected cells (− 1.3 fold changes). Homeobox genes also have an inevitable role in development and cell fate through recruitment of transcriptional co-repressor or co-activators to the promoter sequence of target genes. Therefore, in present study we assessed the expression of MEIS1 as one of the HOX family members in KCTD12 transfected cells. In contrast with the other factors, we have observed a significant positive correlation between KCTD12 and MEIS1 in which the KCTD12 up regulated the MEIS1 (8.9 fold changes) (Fig. 4).

### ABC transporters

**Fig. 5:**
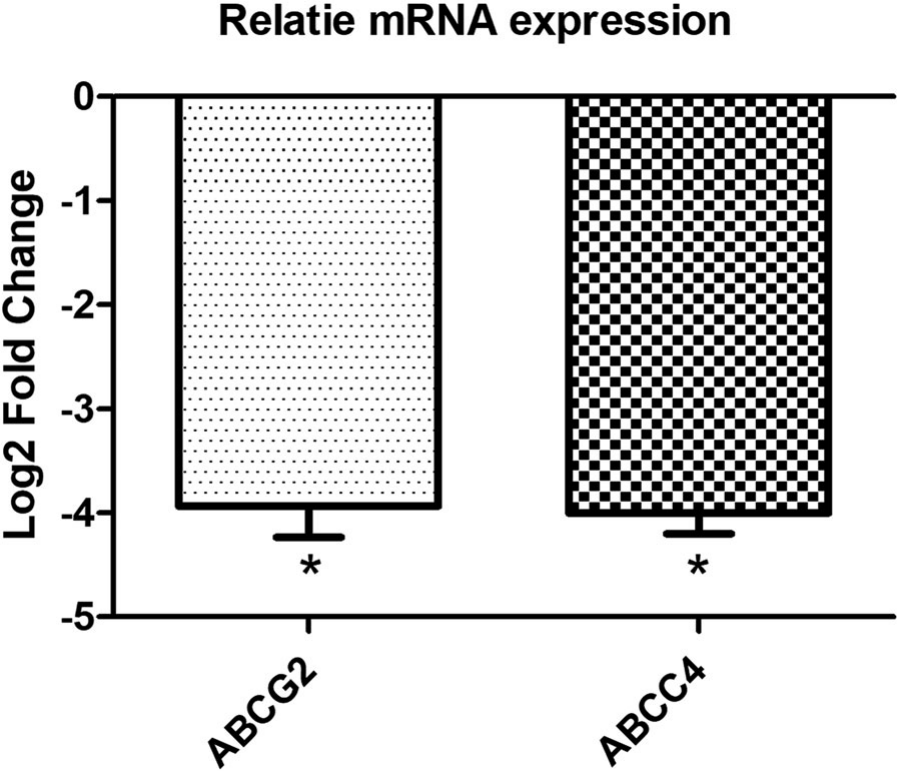
Relative mRNA expression of ABC transporters in KCTD12 transfected in comparison with non-transfected cells

Tumor relapse is one of the main problems during the cancer treatment which is directly related to the drug resistance in tumor cells. It has been shown that the ABC transporters are the first line factors in tumor drug resistance. Therefore, we have assessed the probable correlation between KCTD12 and ABCC4 and ABCG2. Interestingly, KCTD12 down regulated the expression of ABCC4 and ABCG2 (− 4.00 and − 3.93 fold changes, respectively), highlighting the probable role of KCTD12 in drug resistance in ESCC patients via these transporters (Fig. 5).

### Role of KCTD12 in migration and 5FU resistance

**Fig. 6:**
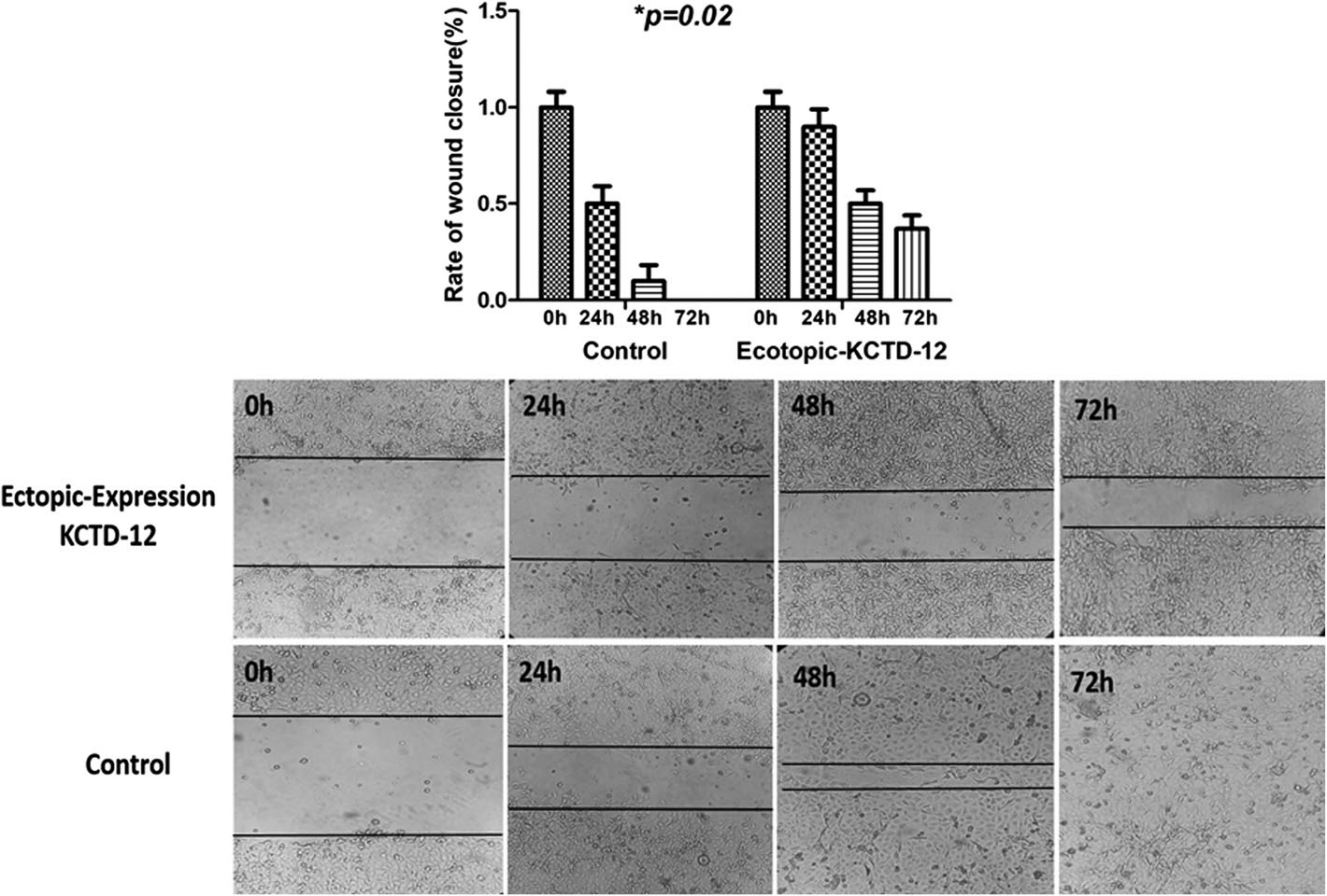
Migration assay in KCTD12 transfected in comparison with non-transfected KSE30

**Fig. 7:**
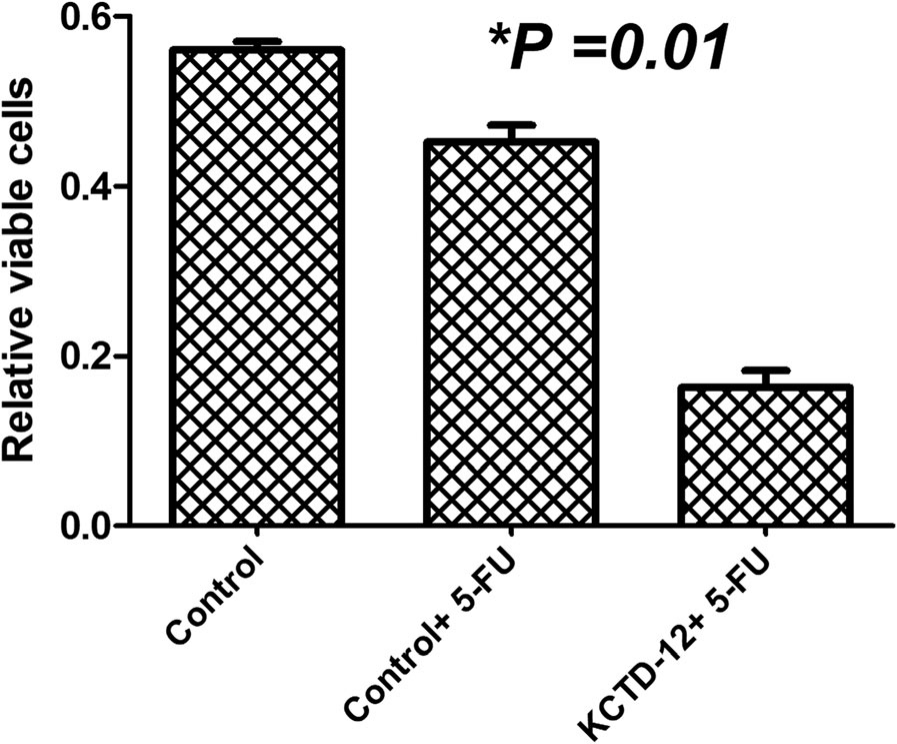
Drug resistance assay for the 5FU in KCTD12 transfected cells in comparison with control

Scratch assay was performed to assess the probable role of KCTD12 in migration of ESCC cells. A significant decrease in cell migration was observed in KCTD12 transfected cells, compared with non-transfected cells (*p* = 0.02) (Fig. 6). We observed that the ectopic expression of KCTD12, significantly decreased the 5FU resistance in KCTD12-5FU in comparison with the control non-transfected cells (*p* = 0.01) (Fig. 7).

## Discussion

**Fig. 8:**
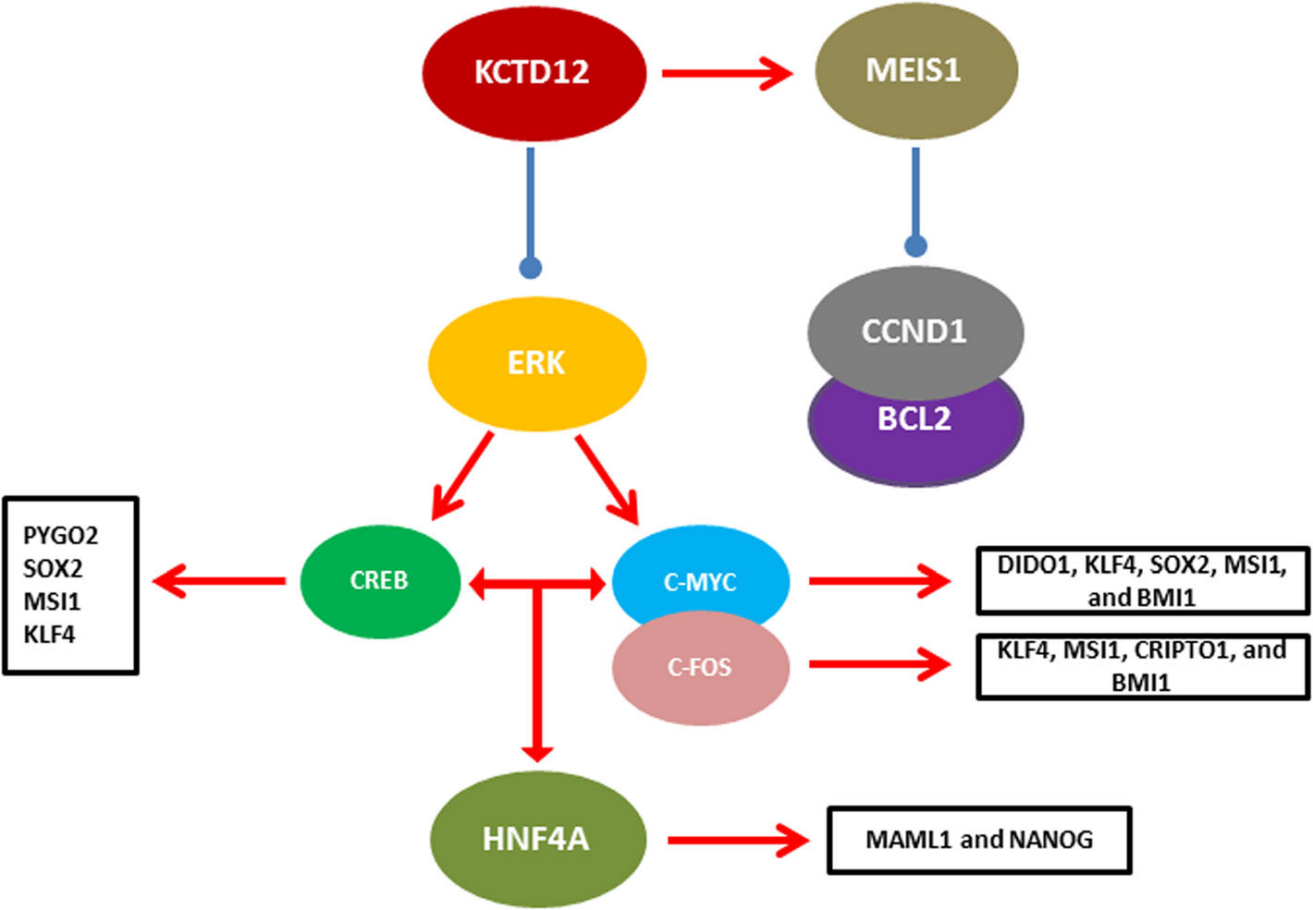
Probable correlations between KCTD12, NOTCH/WNT signaling pathways, chromatin remodelers, and HOX genes in esophageal squamous cell carcinoma

The KCTD proteins are involved in various cellular biological processes such as proliferation, differentiation, and apoptosis [30, 31]. KCTD12 over expression in colorectal cancer repressed stemness through down regulation of CD44, CD133, and CD29 [15]. In OCM-1 cell, KCTD12 ectopic expression also suppressed cell growth via down regulation of VEGF-A, VEGF-C, Snail, and Slug [32]. Therefore, regarding the participation of snail and slug as the major members in EMT process, the KCTD12 is a suppressor of EMT process. In this study, we assessed the role of KCTD12 on expression patterns of different pathways and cellular processes such as NOTCH/WNT signaling pathways, chromatin remodelers, and HOX genes in esophageal squamous cell carcinoma (Fig. 8). Our results showed that KCTD12 has an inhibitory role on NOTCH and WNT signaling pathways and their regulators such as CRIPTO1 and MSI1. In this regard it requires a mediator for exertion of its role which can be done by the chromatin remodelers. Indeed, the transcription machineries need an open chromatin structure to bind to DNA and perform their job. We have shown that the KCTD12 have a correlation with SMARCA4 and BMI1 as important chromatin remodelers. Therefore, it seems that the KCTD12 exerts its inhibitory role via the inhibition of chromatin remodelers. This is the first correlation of K^+^ ion exchange system and chromatin remodeling process. It has been shown that the KCTD suppresses the stemness by ERK inhibition [15]. ERK signaling pathway exerts its role through several important transcription factors such as CREB, C-FOS, and C-MYC. MSI1 and CRIPTO1 as the regulators of WNT/NOTCH pathways have specific sequences in their promoters for C-FOS binding. MSI1 is an inhibitor for the NUMB which is the suppressor of NOTCH pathways. Therefore, lack of MSI1 expression will result in activation of NUMB and MAML1 under expression as the main regulator of NOTCH transcription machinery. KLF4, PYGO2, and SOX2 have also specific sequences for CREB in their promoter sequences. BMI1 promoter sequence has also several binding sequence for C-FOS and C-MYC as the main targets of ERK pathway. Therefore, up regulation of KCTD will result in down regulation of such factors through the inhibition of ERK pathway. In contrast with the other markers, we have observed a significant MEIS1 over expression that can be related to the inhibitory role of this factor through the suppression of CCND1 and BCL2 [33]. DIDO1 can exert its tumor suppressor role through apoptosis induction and its oncogenic role via the activation of NANOG. In present study KCTD12 down regulated the DIDO1 expression highlighting the oncogenic role of this factor in ESCC. Moreover, DIDO1 as a negative mediator also participate in down regulation of NANOG through the KCTD12. In the case of EGFR, we have previously reported that the EGFR as one of the WNT target genes has a positive feedback with b-catenin through stabilization of b-catenin and its translocation into the nucleus [7, 8, 34]. This study also assessed the probable role of KCTD12 in cell motility and data showed that this factor can decrease the cell movement which can be related to the TWIST1. Therefore, KCTD12 may exert its anti-migratory role through TWIST1. We have shown that the KCTD12 significantly decreases the 5FU resistance in KYSE30 which is probably using the down regulation of ABCC4 and ABCG2.

## Conclusions

We have shown that KCTD12 acts as a tumor suppressor in ESCC through different signaling pathways and chromatin remodeling. Our results showed that KCTD12 decreases the cell migration of ESCC cells through TWIST1 and can be introduced as a therapeutic marker against the EMT process and tumor relapse. Moreover, role of KCTD12 in 5FU resistance introduces that as an efficient marker for the epigenetic targeted therapy of esophageal squamous cell carcinoma.

## References

[1] Ferlay J (2010). Estimates of worldwide burden of cancer in 2008: GLOBOCAN 2008. Int J Cancer.

[2] Kamangar F, Dores GM, Anderson WF (2006). Patterns of cancer incidence, mortality, and prevalence across five continents: defining priorities to reduce cancer disparities in different geographic regions of the world. J Clin Oncol.

[3] Mahboubi E (1973). Oesophageal cancer studies in the Caspian littoral of Iran: the Caspian cancer registry. Br J Cancer.

[4] Dipetrillo T (2012). Neoadjuvant paclitaxel poliglumex, cisplatin, and radiation for esophageal cancer: a phase 2 trial. Am J Clin Oncol.

[5] van Hagen P (2012). Preoperative chemoradiotherapy for esophageal or junctional cancer. N Engl J Med.

[6] Abbaszadegan MR, Moghbeli M (2018). Role of MAML1 and MEIS1 in Esophageal Squamous Cell Carcinoma Depth of Invasion. Pathol Oncol Res.

[7] Moghbeli M (2013). Association of PYGO2 and EGFR in esophageal squamous cell carcinoma. Med Oncol.

[8] Moghbeli M (2016). Correlation of Wnt and NOTCH pathways in esophageal squamous cell carcinoma. J Cell Commun Signal.

[9] Moghbeli M (2014). Clinicopathological sex- related relevance of musashi1 mRNA expression in esophageal squamous cell carcinoma patients. Pathol Oncol Res.

[10] Moghbeli M (2016). Correlation between Meis1 and Msi1 in esophageal squamous cell carcinoma. J Gastrointest Cancer.

[11] Resendes BL (2004). Isolation from cochlea of a novel human intronless gene with predominant fetal expression. J Assoc Res Otolaryngol.

[12] Cathomas F (2015). Altered emotionality and neuronal excitability in mice lacking KCTD12, an auxiliary subunit of GABAB receptors associated with mood disorders. Transl Psychiatry.

[13] Suehara Y (2008). Pfetin as a prognostic biomarker of gastrointestinal stromal tumors revealed by proteomics. Clin Cancer Res.

[14] Zhong Y (2017). KCTD12 promotes tumorigenesis by facilitating CDC25B/CDK1/aurora A-dependent G2/M transition. Oncogene.

[15] Li L (2016). KCTD12 regulates colorectal Cancer cell Stemness through the ERK pathway. Sci Rep.

[16] Kang HJ (2006). Differentially expressed proteins in gastrointestinal stromal tumors with KIT and PDGFRA mutations. Proteomics.

[17] Canettieri G (2010). Histone deacetylase and Cullin3-REN(KCTD11) ubiquitin ligase interplay regulates hedgehog signalling through Gli acetylation. Nat Cell Biol.

[18] De Smaele E (2011). Identification and characterization of KCASH2 and KCASH3, 2 novel Cullin3 adaptors suppressing histone deacetylase and hedgehog activity in medulloblastoma. Neoplasia.

[19] Koch U, Lehal R, Radtke F (2013). Stem cells living with a Notch. Development.

[20] Liu J (2010). Notch signaling in the regulation of stem cell self-renewal and differentiation. Curr Top Dev Biol.

[21] Bedogni B (2008). Notch1 is an effector of Akt and hypoxia in melanoma development. J Clin Invest.

[22] Reedijk M (2005). High-level coexpression of JAG1 and NOTCH1 is observed in human breast cancer and is associated with poor overall survival. Cancer Res.

[23] Santagata S (2004). JAGGED1 expression is associated with prostate cancer metastasis and recurrence. Cancer Res.

[24] South AP, Cho RJ, Aster JC (2012). The double-edged sword of Notch signaling in cancer. Semin Cell Dev Biol.

[25] Choi JD, Lee JS (2013). Interplay between epigenetics and genetics in Cancer. Genomics Inform.

[26] Clapier CR, Cairns BR (2009). The biology of chromatin remodeling complexes. Annu Rev Biochem.

[27] Jones PA, Baylin SB (2007). The epigenomics of cancer. Cell.

[28] Shain AH (2012). Convergent structural alterations define SWItch/sucrose NonFermentable (SWI/SNF) chromatin remodeler as a central tumor suppressive complex in pancreatic cancer. Proc Natl Acad Sci U S A.

[29] Taleb S (2014). HES1 as an independent prognostic marker in esophageal squamous cell carcinoma. J Gastrointest Cancer.

[30] Faryna M (2012). Genome-wide methylation screen in low-grade breast cancer identifies novel epigenetically altered genes as potential biomarkers for tumor diagnosis. FASEB J.

[31] Mancarelli MM (2010). The tumor suppressor gene KCTD11REN is regulated by Sp1 and methylation and its expression is reduced in tumors. Mol Cancer.

[32] Luo L (2017). Lentiviral-mediated overexpression of KCTD12 inhibits the proliferation of human uveal melanoma OCM-1 cells. Oncol Rep.

[33] Zhu J (2017). MEIS1 inhibits clear cell renal cell carcinoma cells proliferation and in vitro invasion or migration. BMC Cancer.

[34] Moghbeli M (2016). Role of Msi1 and PYGO2 in esophageal squamous cell carcinoma depth of invasion. J Cell Commun Signal.

